# Histological Characteristics and Early-Stage Diagnosis Are Associated With Better Survival in Young Patients With Epithelial Ovarian Cancer: A Retrospective Analysis Based on Surveillance Epidemiology and End Results Database

**DOI:** 10.3389/fonc.2020.595789

**Published:** 2020-12-23

**Authors:** Yue Huang, Xiu Ming, Bingjie Li, Zhengyu Li

**Affiliations:** ^1^ Department of Gynecology and Obstetrics, West China Second University Hospital, Sichuan University, Chengdu, China; ^2^ Key Laboratory of Birth Defects and Related Diseases of Women and Children (Sichuan University), Ministry of Education, Chengdu, China; ^3^ Department of Biotherapy, Cancer Center, State Key Laboratory of Biotherapy, West China Hospital, Sichuan University, and Collaborative Innovation Center, Chengdu, China

**Keywords:** Surveillance Epidemiology and End Results database, AJCC stage, survival, histological characteristics, epithelial ovarian cancer

## Abstract

**Purpose:**

To analyze the potential prognostic factors of epithelial ovarian cancer (EOC) in women aged under 35 compared to those aged 60–79.

**Methods:**

Cases were retrospectively obtained from SEER database. Clinical characteristics, such as race, histological type, AJCC stage, laterality of tumors, CA125 results, and surgical strategies, were analyzed in < 35 years group and 60–79 years group. Kaplan-Meier survival curves were used to evaluate overall survival (OS) and cause-specific survival (CSS). Cox proportional hazard model was used to identify the predictors for CSS.

**Results:**

Sixteen thousand eight hundred forty-seven EOC patients diagnosed in 2004–2015 were identified from SEER database, with 1,015 aged under 35 and 15,833 aged 60–79. In < 35 years group, mucinous (32.2%) was the most common histological type, followed by high-grade serous (26.6%) and endometrioid (18.3%), while in 60–79 years group, high-grade serous (68.3%) represented the leading histological type. Most young women were diagnosed at stage I (57.7%), while most old women were diagnosed at stage (48.1%). Both 5-year OS and 5-year CSS were higher in < 35 years group (5-year OS: 76.00% vs 40.18%, p < 0.001; 5-year CSS: 83.56% vs 55.18%, p < 0.001). The multivariate analysis identified histological type and stage as prognostic factors for CSS in both groups. Endometrioid represented a positive predictor for CSS, while carcinosarcoma and malignant Brenner were related to a worse CSS. (< 35 years group: carcinosarcoma vs endometrioid: HR 5.630, p=0.024; malignant Brenner vs endometrioid: HR 4.005, p < 0.001; 60–79 years group: carcinosarcoma vs endometrioid: HR 3.606, p < 0.001; malignant Brenner vs endometrioid: HR 2.291, p < 0.001). Tumors laterality, CA125 levels, surgery and lymphadenectomy failed to be associated with the CSS in < 35 years group, while found to be independent risk factors in 60–79 years group.

**Conclusion:**

EOC women aged under 35 had a better survival outcome over EOC women aged 60–79, owing to high proportion of endometrioid and mucinous types in histology, as well as early-stage diagnosis. Identification of histological types and gene profiles should be underscored in young EOC patients.

## Introduction

Ovarian cancer (OC), one of the most lethal gynecological cancers, caused 295,414 new cases and 184,799 deaths across the world in 2018 (1). Although the mortality has decreased due to the improvement of treatment strategies in the past 50 years, the 5-year survival rate is still under 50% because of late diagnosis (2). Epithelial ovarian cancer (EOC), which includes several subtypes with distinctive pathological and clinical features, takes up the majority of OC patients (3). EOC has the highest incidence rate in women in their 6^th^ to 7^th^ decades (4), while is rarely seen in young women, with 1.1% in aged under 25, and 4.1% in aged under 30 (5). Several studies (5–11) have focused on this small group of patients and found that they were associated with distinct patterns of clinicopathological characteristics and a higher overall survival rate. However, few studies included a large population, and some did not analyze the surgical strategies and the associated outcomes in the patient group. Women’s fertility rate sharply decreases after age of 35 (12). Thus, women aged under 35 represent a special group to be considered in many GYN/OB diseases. By retrospectively analyzing the data obtained from the National Cancer Institute’s Surveillance, Epidemiology, and End Results (SEER) program, the current study aims to conduct a thorough analysis including clinicopathologic characteristics, surgical strategies and their relationship with the survival outcome in women aged under 35 compared to women aged 60–79, hoping to provide some helpful evidence for young EOC patients’ counseling and treatment strategies selection.

## Methods

### Study Population

Women with EOC under the age of 35 were included in < 35 years group, while women with EOC aged between 60 and 79 were identified as 60–79 years group for comparison.

### Data Extraction

**Figure 1:**
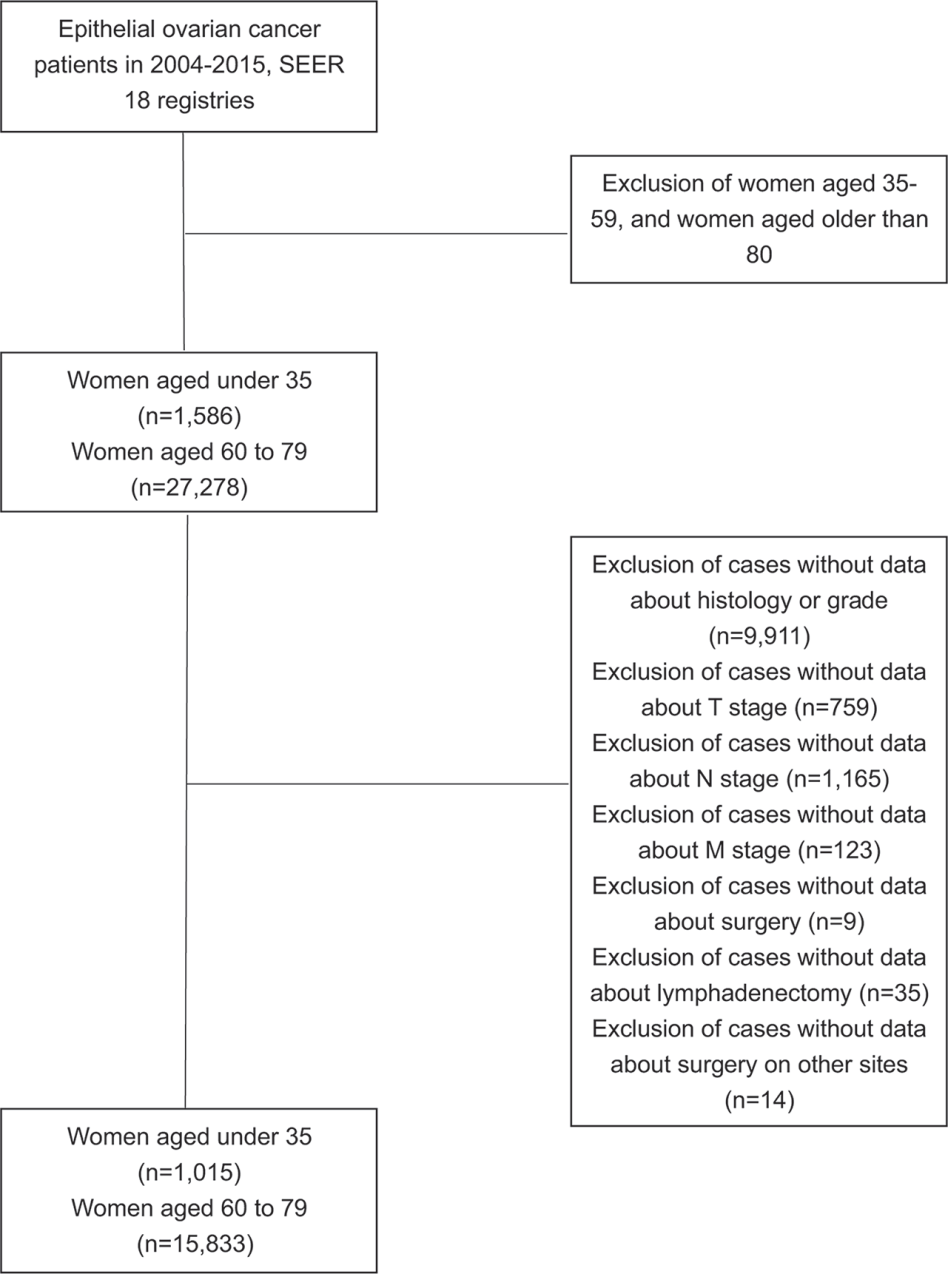
Flow diagram for patient inclusion.

Data in this study was extracted from the National Cancer Institute’s Surveillance, Epidemiology, and End Results (SEER) program released in November 2016. Women diagnosed with EOC (primary site: C56.9 Ovary) from 2004 to 2015 with the age range of 0–34 or 60–79 were identified. Parameters extracted included demographic, clinicopathological, treatment and survival information. The histological classification was identified according to 2014 WHO EOC histological types and a prior population-based study (13). The histological results were collected from pathology laboratories according to the standards issued by the North American Association of Central Cancer Registries (NAACR). Exclusion criteria were cases with missing information on histological types, AJCC stage, surgery intervention, lymph nodes removement during operation or surgery on remote sites (**Figure 1**). Surgical methods were classified into no surgery performed, unilateral salpingo-oophorectomy (USO) or bilateral salpingo-oophorectomy (BSO) (without hysterectomy), USO or BSO (with hysterectomy), USO or BSO (NOS), debulking or cytoreductive surgery (NOS), pelvic exenteration, and others.

### Statistical Analysis

Median age of each group was calculated by independent samples t-test, and the rest variables were evaluated by chi-squared test, Fisher exact test, or post hoc test. Kaplan-Meier survival curves and log-rank test with 95% confidence intervals (CIs) were applied to evaluate the outcomes between the two groups. Risk factors were analyzed using Cox proportional hazards regression model with 95%CI. We considered p < 0.05 as statistically significant. For the post hoc test, the adjusted standardized value > 3 was regarded statistically significant. Data extraction was completed in SEER*Stat, and all analyses were performed using the SPSS Statics software, version 25.0 (IBM Corporation, NY, USA).

## Results

### Included Patient Characteristics

As shown in **Table 1**, 16,847 EOC patients were extracted from the SEER database, with 1,015 patients younger than 35 years, and 15,833 aged 60–79. Mucinous (32.2%) was the most common histological type in the < 35 years group, followed by high-grade serous (26.6%) and endometrioid (18.8%) tumors, while high-grade serous (68.3%) was most commonly observed type in the 60–79 years group. As for AJCC stage, over half of the young women (57.7%) were diagnosed at Stage I, while nearly half of the old women (48.1%) were diagnosed at stage III. The association between histological type and AJCC stage were analyzed in both groups (**Table S1**). Patients who were diagnosed at stage III were more likely to have high-grade serous tumors (56.3% in < 35 years group, 56.4% in 60–79 years group) and low-grade serous tumors (60.3% in < 35 years group, 47.4% in 60–79 years group), while those diagnosed at stage I were more likely to have endometrioid tumors (80.1% in < 35 years group, 70.5% in 60–79 years group) and mucinous tumors (85.3% in < 35 years group, 65.4% in 60–79 years group).The CA125 level elevated in most patients in both group (55.7% and 71.6%, respectively). The statistical significances of clinicopathological characteristics were evaluated by post hoc test (**Table S2**).

**Table 1:** Clinicopathological characteristics of young and old patients with epithelial ovarian cancer in 2004–2015, SEER 18 registries.

		Patients <35 years (n=1,015)	Patients ≥60 and ≤79 years (n=15,833)	P-value
Age at diagnosis		28.42±4.87	68.16±5.57	<0.001
Race/ethnicity	White^a^	797(78.5%)	13,797(87.1%)	<0.001
	Black	61(6.0%)	996(6.3%)	
	Asian or Pacific Islander^a^	147(14.5%)	952(6.0%)	
	Others or unknown	10(1.0%)	88(0.6%)	
Histology ICD-O3	High-grade serous^a^	270(26.6%)	10,809(68.3%)	<0.001
	Low-grade serous^a^	78(7.7%)	306(1.9%)	
	Endometrioid^a^	186(18.3%)	1,089(6.9%)	
	Mucinous^a^	327(32.2%)	654(4.1%)	
	Clear cell	37(3.6%)	728(4.6%)	
	Carcinosarcoma^a^	5(0.5%)	300(1.9%)	
	Malignant Brenner	52(5.1%)	1,053(6.7%)	
	Mixed	60(5.9%)	894(5.6%)	
Laterality	Right-origin primary^a^	370(36.5%)	4,291(27.1%)	<0.001
	Left-origin primary^a^	384(37.8%)	4,144(26.2%)	
	Paired site, but no information of laterality^a^	13(1.3%)	600(3.8%)	
	Bilateral, single primary^a^	248(24.4%)	6,693(42.3%)	
	Only one side-side unspecified	0	105(0.7%)	
T	T0	0	19(0.1%)	<0.001*
	T1^a^	612(60.3%)	3,341(21.1%)	
	T2^a^	95(9.4%)	2,224(13.1%)	
	T3^a^	308(30.3%)	10,249(63.5%)	
N	N0	845(83.3%)	11,878(75.0%)	<0.001
	N1	170(16.7%)	3,955(25.0%)	
M	M0	929(91.5%)	12,323(77.8%)	<0.001
	M1	86(8.5%)	3,510(22.2%)	
AJCC Stage	I^a^	586(57.7%)	3,082(19.5%)	<0.001
	II^a^	72(7.1%)	1,621(10.2%)	
	III^a^	271(26.7%)	7,620(48.1%)	
	IV^a^	86(8.5%)	3,510(22.2%)	
CA125	Positive/elevated^a^	565(55.7%)	11,337(71.6%)	<0.001*
	Borderline	3(0.3%)	17(0.1%)	
	Negative/normal^a^	154(15.2%)	1,327(8.4%)	
	Results unknown	6(0.6%)	135(0.9%)	
	Test not done^a^	131(12.9%)	959(6.1%)	
	Unknown if the test did or not	156(15.4%)	2,058(13.0%)	

*Fisher test.

a Statistical significance between the two groups by post hoc test (**Table S2**).

### Treatment Strategies

Only 1.1% young women and 3.6% old women did not have surgery. In < 35 years group, more patients underwent uterine-preserving surgery than those in 60–79 years group (43.7% vs 10.1%), while more old women underwent debulking or cytoreductive surgery than their young counterparts (47.0% vs 21.3%). Besides, more young women underwent lymphadenectomy than old women (68.4% vs 55.2%, p < 0.001). There was no significance in the tendency of surgery on distant sites in the two groups (**Table 2**)

**Table 2:** Surgery details of young and old patients with epithelial ovarian cancer in 2004–2015, SEER 18 registries.

		Patients <35 years (n=1,015)	Patients ≥60 and ≤79 years (n=15,833)	P-value
Surgery	USO or BSO, without hysterectomy	444(43.7%)	1604(10.1%)	<0.001
	USO or BSO, with hysterectomy	297(29.3%)	5454(34.4%)	
	USO or BSO, NOS	33(3.3%)	330(2.1%)	
	debulking; cytoreductive surgery, NOS	216(21.3%)	7437(47.0%)	
	pelvic exenteration	13(1.3%)	394(2.5%)	
	no surgery	11(1.1%)	567(3.6%)	
	others	1(0.1%)	47(0.3%)	
Lymphadenectomy	Lymphadenectomy not done	321(31.6%)	7,087(44.8%)	<0.001
	Lymphadenectomy done	694(68.4%)	8,746(55.2%)	
Surgery on other sites	Surgery on other regions not done	853(84.0%)	13,587(85.8%)	0.117
	Surgery on other regions	162(16.0%)	2.246(14.2%)	
Reason of surgery not done	Surgery performed	1,004(98.9%)	15,266(96.4%)	<0.001*
	Surgery not recommended/contraindications	8(0.8%)	494(3.1%)	
	Patients died before planned surgery	0	9(0.1%)	
	Patients or their relatives refused	2(0.2%)	12(0.1%)	
	Unknown	1(0.1%)	52(0.3%)	

*Fisher test.

BSO, bilateral salpingo-oophorectomy; USO, unilateral salpingo-oophorectomy.

### Survival Outcomes

**Figure 2:**
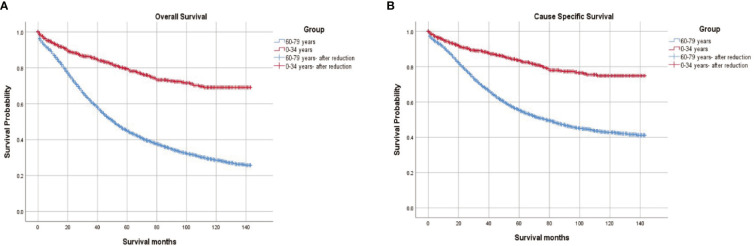
**(A)** Overall survival and **(B)** Cause-specific survival in women aged <35 and women aged 60 to 79 diagnosed with epithelial ovarian cancer.

The survival outcomes of the two groups were illustrated in **Figure 2**. Compared to women aged between 60 and 79 years, young women had a better 5-year overall survival (OS) (76.00% vs 40.18%, p < 0.001) and cause-specific survival (CSS) (83.56% vs 55.18%, p < 0.001).

**Figure 3:**
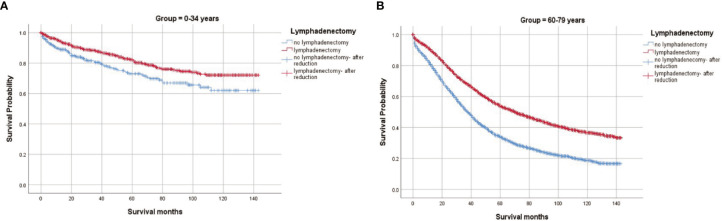
Cause-specific survival by lymphadenectomy in women aged <35 and women aged 60 to 79 diagnosed with epithelial ovarian cancer. **(A)** Lymphadenectomy in women aged <35; **(B)** Lymphadenectomy in women aged 60.

CSS by lymphadenectomy were further identified (**Figure 3**). In < 35 years group, lymphadenectomy did not indicate a significantly better outcome (5-year CSS 84.21% vs 82.12%, p=0.318). In 60–79 years group, however, those who had their lymph nodes removed had a much higher CSS rate (63.22% vs 44.78%, p < 0.001). Furthermore, we analyzed the survival curves on lymphadenectomy by stages in both groups. In < 35 years group, lymphadenectomy made no significant differences for patients diagnosed at stage I/II (93.19% vs 94.72%, p=0.686) or at stage III/IV (64.37% vs 63.19%, p=0.828), while in 60–79 years group, both patients diagnosed at stage I/II (88.49% vs 80.39%, p < 0.001) and those diagnosed at stage III/IV (47.36% vs 35.86%, p < 0.001) benefited from lymphadenectomy (**Figure S1**).

### Risk Factors of Cause-Specific Survival

In the < 35 years group, histological type, laterality of tumors, AJCC stage, level of CA125 before therapies, surgery, and surgery on other sites (all p < 0.001) were risk factors for CSS in univariate survival analysis (**Table 3**). In multivariate analysis, however, only histological type and AJCC stage remained as independent prognostic factors. Compared to endometrioid histological type, carcinosarcomas (Hazard ratio (HR) 5.630 95%CI 1.256, 25.226, p=0.024) and malignant Brenner tumors (HR 4.005 95%CI 1.880, 8.531, p < 0.001) were related to a worse CSS. As for the AJCC stage, the risk increased as the stage advanced (T2 versus T1, HR 2.896, 95%CI 1.381, 5.962, p=0.005; T3 versus T1 HR 8.724, 95%CI 5.355, 14.213, p < 0.001; T4 versus T1 HR 26.856, 95%CI 16.009, 45.054, p < 0.001).

**Table 3:** Univariate analysis by Cox regression for CSS in both young and old women group with epithelial ovarian cancer in 2004–2015, SEER 18 registries.

		Patients <35 years (n=1,015)		Patients ≥60 and ≤79 years (n=15,833)	
		5-year CSS	P	5-year CSS	P
Race/ethnicity	White	83.27%	0.436	55.37%	<0.001
	Black	87.51%		45.90%	
	Asian or Pacific Islander	83.63%		62.62%	
	Others or unknown	87.50%		54.85%	
Histology ICD-O3	High-grade serous	74.31%	<0.001	49.79%	<0.001
	Low-grade serous	87.76%		78.96%	
	Endometrioid	90.83%		90.36%	
	Mucinous	90.28%		73.92%	
	Clear cell	86.65%		71.92%	
	Carcinosarcoma	60.00%		36.84%	
	Malignant Brenner	61.71%		43.75%	
	Mixed	77.26%		59.97%	
Laterality	Right-origin primary	88.00%	<0.001	65.51%	<0.001
	Left-origin primary	89.27%		67.11%	
	Paired site, but no information of laterality	38.24%		33.76%	
	Bilateral, single primary	69.86%		43.30%	
	Only one side-side unspecified			42.27%	
AJCC Stage	I	94.68%	<0.001	90.99%	<0.001
	II	85.85%		76.44%	
	III	72.39%		46.51%	
	IV	36.79%		31.02%	
CA125	Positive/elevated	78.41%	<0.001	50.15%	<0.001
	Borderline	100%		69.84%	
	Negative/normal	91.20%		81.72%	
	Results unknown	83.33%		56.04%	
	Test not done	90.78%		62.45%	
	Unknown if the test did or not	88.04%		61.09%	
Surgery	Surgery not performed	26.26%	<0.001	16.91%	<0.001
	FSS/no TAH&BSO	92.94%		65.71%	
	non-FSS/TAH&BSO	86.08%		75.60%	
	FSS, NOS/TAH or BSO, NOS	89.82%		66.72%	
	Debulking	67.33%		44.54%	
	Pelvic exenteration	0.00%		39.73%	
	Surgery performed, but methods unknown	100.00%		50.34%	
Lymphadenectomy	Lymphadenectomy not done	82.12%	0.318	44.48%	<0.001
	Lymphadenectomy done	84.21%		63.22%	
Surgery on other sites	Surgery on other regions not done	86.22%	<0.001	56.44%	<0.001
	Surgery on other regions	68.67%		47.22%	

HR, hazard ratio; CSS, cause-specific survival.

In the 60–79 years group, race, histological type, laterality of tumors, AJCC stage, level of CA125 before therapies, surgery, lymphadenectomy, and surgery on other sites (all p < 0.001) were all associated with CSS. Only surgery on other sites (p=0.715) was excluded in the multivariate survival analysis. Bilateral tumors represented risk factors for CSS (right-origin primary tumor as a reference, paired sites, NOS HR 1.210, 95%CI 1.051, 1.393, p=0.008; bilateral, single primary, HR 1.228, 1.147, 1.316, p<0.001) (**Table 4**).

**Table 4:** Multivariate analysis by Cox regression for CSS in both young and old women group with epithelial ovarian cancer in 2004–2015, SEER 18 registries.

		Patients <35 years (n=1,015)			Patients ≥60 and ≤79 years (n=15,833)		
		HR	95%CI	P	HR	95%CI	P
Race/ethnicity	White	not included			1.000		0.000
	Black				1.235*	1.119,1.364	0.000
	Asian or Pacific Islander				0.857*	0.758,0.969	0.014
	Others or unknown				1.094	0.791,1.513	0.586
Histology ICD-O3	Endometrioid	1.000		0.000	1.000		0.000
	High-grade serous	0.844	0.440,1.620	0.610	1.958*	1.578,2.430	0.000
	Low-grade serous	0.466	0.192,1.130	0.091	1.105	0.797,1.532	0.548
	Mucinous	1.505	0.780,2.904	0.223	3.177*	2.443,4.132	0.000
	Clear cell	1.678	0.595,4.732	0.328	2.780*	2.139,3.612	0.000
	Carcinosarcoma	5.630*	1.256,25.226	0.024	3.606*	2.762,4.706	0.000
	Malignant Brenner	4.005*	1.880,8.531	0.000	2.291*	1.817,2.887	0.000
	Mixed	1.621	0.726, 3.617	0.238	2.090*	1.642,2.660	0.000
Laterality	Right-origin primary			0.074	1.000		0.000
	Left-origin primary			0.242	0.992	0.914,1.078	0.854
	Paired site, but no information of laterality			0.059	1.210*	1.051,1.393	0.008
	Bilateral, single primary			0.156	1.228*	1.147,1.316	0.000
	Only one side-side unspecified				1.250	0.948,1.647	0.114
AJCC Stage	I	1.000		0.000	1.000		0.000
	II	2.869*	1.381,5.962	0.005	2.433*	2.050,2.888	0.000
	III	8.724*	5.355,14.213	0.000	5.448*	4.706,6.307	0.000
	IV	26.856*	16.009,45.054	0.000	7.861*	6.758,9.143	0.000
CA125	Negative/normal			0.163	1.000		0.000
	Positive/elevated			0.036	1.571*	1.363,1.810	0.000
	Borderline			0.593	1.179	0.438,3.174	0.744
	Results unknown			0.151	1.838*	1.362,2.482	0.000
	Test not done			0.063	1.448*	1.212,1.730	0.000
	Unknown if the test did or not			0.510	1.542*	1.317,1.806	0.000
Surgery	FSS/no TAH&BSO			0.121	1.000		0.000
	non-FSS/TAH&BSO			0.746	0.863*	0.751,0.991	0.037
	FSS, NOS/TAH or BSO, NOS			0.519	0.951	0.855,1.056	0.346
	Debulking			0.679	1.115*	1.013,1.227	0.027
	Pelvic exenteration			0.033	1.135	0.959,1.344	0.141
	Surgery performed, but methods unknown			0.543	1.066	0.646,1.757	0.803
	Surgery not performed			0.078	2.501*	2.147,2.913	0.000
Lymphadenectomy	Lymphadenectomy done	not included			1.000		
	Lymphadenectomy not done				1.339*	1.268,1.414	0.000
Surgery on other sites	Surgery on other regions not done			0.172			0.715
	Surgery on other regions						

HR, hazard ratio, CI, confidence interval.

## Discussion

This population-based study retrospectively analyzed different patterns of clinicopathological characteristics, treatment and outcomes between women with EOC aged under 35, those aged 60–79. Mucinous tumors and stage I represented the most common histological type and AJCC stage observed in young women, respectively, and both of them indicated better survival outcomes. For old women, however, high-grade serous tumors and stage III were most commonly seen, and they indicated worse survival outcomes. Histological type and AJCC stage were prognostic factors for CSS in both groups. For EOC patients aged 60–79, laterality, CA125 levels, surgery techniques, and lymphadenectomy were risk factors for CSS only. To our knowledge, the current study is the first study analyzing clinicopathological characteristics, treatment and survival outcomes in a large group of young EOC patients.

Mucinous (32.2%) represented the most common histological type in women aged under 35, followed by high-grade serous (26.6%), and endometrioid (18.3%) tumors. Our results are similar to a Japanese population-based study that demonstrated mucinous (36.7%) as the most prevalent histological type, followed by clear-cell (28.7%) and endometrioid (19.6%) in EOC women aged 40 and younger (14). However, other retrospective studies (6, 8–10, 13) found that serous histological type took up the largest portion in EOC women aged under 35 or 40. The difference could be explained by the following reasons. Firstly, most studies did not apply 2014 WHO EOC histology classification, which divides serous histological type into high-grade and low-grade. Secondly, the different age ranges and different ethnicity of included patients could also cause the difference. It should be noted that some included patients with mucinous histological type might have mucinous carcinomas that originated from the gastrointestinal tract, because mucinous carcinomas originated from different sites share very similar pathological characteristics (15). Currently, there are no immunohistochemical (IHC) algorithms for mucinous EOC, and an accurate diagnosis of primary mucinous EOC warrants a combination of the IHC, biomarkers and imaging results (16). Shimada et al. reviewed the pathological results of mucinous EOC patients and found only 33.9% were diagnosed with mucinous invasive carcinomas (17). It is agreed that with improved histopathology techniques and a greater understanding of mucinous carcinomas, the incidence of mucinous EOC drops to 3% (18). Therefore, the percentage of mucinous EOC in both < 35 years and 60–79 years group would be lower than the current results.

In both < 35 years group and 60–79 years group, endometrioid, mucinous, low-grade serous, and clear cell tumors indicated a higher 5-year CSS, while carcinomas, malignant Brenner tumors, and high-grade serous indicated lower 5-year CSS in the univariate survival analysis. The results are similar to the study of Aihua Lan (19), which is based on a SEER database without age stratification. In the multivariate analysis, however, mucinous was related to a poorer survival rate in 60–79 years group. In < 35 years group, although there was no statistical significance, the hazard rate of mucinous histological type was 1.505. The difference of mucinous-associated survival outcomes could result from several factors. Firstly, as we mentioned above, patients with mucinous carcinomas metastasized from the gastrointestinal tract were also included in our study and this group of patients would have much worse survival outcomes. Secondly, mucinous EOC diagnosed at an early stage had favorable prognosis, while those diagnosed at a late stage and recurrent tumors had poor survival outcomes because of the insensitivity to chemotherapy (13, 20–23). Increasing studies focused on unfolding the genetic secret of mucinous EOC in recent years. The mutation of KRAS protein, which is associated with the RAS/RAF/MARK pathway, might play an important role in the beginning event of mucinous EOC (24, 25). The amplification of HER2 and p53 genes mutation were also reported in patients with mucinous EOC, and both of them were associated with the malignant transformation in the stepwise progression of mucinous EOC (15, 22). Considering the high incidence of mucinous EOC in young women, gene diagnosis for prognosis and therapy consultation could be arranged in this patient group in the future.

The early-stage diagnosis of young women with EOC has been demonstrated in several studies (10, 26, 27). Mucinous and endometrioid histological types, which were commonly observed in young women, often present as localized masses, while high-grade serous tumors, commonly diagnosed in old women, often spread beyond pelvis at diagnosis (27, 28). Moreover, endometriosis-associated ovarian cancer (EAOC) including endometrioid and clear-cell histological types, are more often reported in young women. EAOC usually presents chronic pelvic pain, dyspareunia, dysmenorrhea and infertility, as well as significantly elevated CA125 levels, making them easier to be detected at an early stage (29–31). With the development of advanced molecular techniques, mutations in several genes such as ARID1A, PIK3CA, and CTCF have been found involved in the progression from benign endometriosis to EAOC (32). As a result, it is important for physicians to screen for EAOC in patients with endometriosis, and to initiate management as well as regular monitoring for those with a high risk of EAOC. It is known that positive family history and BRCA1/2 mutation could be detected especially in patients with early-onset EOC, indicating genetics as a key risk factor for young EOC patients (33). Therefore, combing the results of gene profiles with prediction models such as Risk of ovarian malignancy algorithm (ROMA), Copenhagen index (CPH-I), Risk of ovarian cancer algorithm (ROCA), LR2, and the Assessment of Different NEoplasias in the AdneXa (ADNEX) model, would be helpful in early recognition of young women with high risk of EOC and in the further monitoring process (34, 35).

The laterality of tumors was found to be a risk factor for the prognosis of old women, with bilateral tumors indicating a poorer outcome. This could be explained by the findings of Ditto et al. that bilaterality of EOC tumors is associated with lymph node metastases (36). Laterality failed to be a risk factor for young women could be due to the relatively small enrolled number. However, since we lacked the information on chemotherapy, which could act as a confounding factor as a number of old women did not receive chemotherapy due to their poor performance status, resulting in a false–positive result. More literature about the laterality of EOC tumors is thus warranted for further study.

In this study, we have demonstrated that young women did not benefit from lymphadenectomy, while old women with lymphadenectomy had a higher CSS compared to those without lymph nodes removal. The difference might be explained by our finding that young women with EOC were mostly at stage I, while the old were mostly at stage III, which suggests that lymph nodes were more likely to metastasize in old patients; thus those undetected lymph nodes containing metastatic cancer cells in old women would be removed. It could also be explained by the possible selective bias that women underwent lymphadenectomy in the control group might be relatively younger, and with a better health status compared to those without lymphadenectomy. Lymphadenectomy still represents a controversial issue in the surgery for ovarian cancer. The recent LION trial (37) focused on advanced ovarian patients with R0 cytoreductive surgery and negative lymph nodes detected both preoperatively and during the operation. In this patient group, the systemic pelvic and paraaortic lymphadenectomy were not associated with a better OS or progression-free survival (PFS). Similar results were observed in patients with ovarian cancer at early stages (38) and those who underwent lymphadenectomy after primary surgery (39). Due to the limited data on lymph nodes removal such as number, location, postoperative complication, and recurrence rate, we could not get further results. According to the evidence we have, we suggest that for young women there is no need for lymphadenectomy unless lymph nodes detected by radiologic examination or tested positive during the surgery, or fertility-sparing is required.

Some limitations could not be overlooked in the current study. Missing data on histology, stage and surgery might cause selective bias. Secondly, information on residual disease, cytoreduction surgery and neoadjuvant chemotherapy was included since 2010, which restricted us to analyze their roles in survival outcomes in the young patient group. Experience of surgeons, the specific type of lymphadenectomy, the recurrence incidence of EOC, and fertility outcomes of those who underwent uterine preserving surgeries were not included in the SEER database, thus we could not analyze their relationship with the outcomes of EOC patients. Currently, most researchers hold the view that combined biomarkers, instead of single one, are encouraging in the screening process (35, 40).For example, human epididymis protein 4 (HE4) and CA125 are included in ROMA with a sensitivity of 71% and specificity of 88% (41). However, only CA125 results were available in the SEER database.

In summary, women with EOC aged under 35 have higher OS and CSS compared to women aged 60–79, which could be due to a large percentage of mucinous and endometrioid histological types and early-diagnosis in the young EOC women group. Physicians can provide more positive prognosis information for young EOC patients, and the identification of histological types should be underscored in the diagnosis of this patient group. Moreover, gene diagnosis might play an important role in further prognosis assessments and clinical decisions.

## Data Availability Statement

Publicly available datasets were analyzed in this study. This data can be found here: https://seer.cancer.gov/.

## Ethics Statement

Ethical review and approval was not required for the study on human participants in accordance with the local legislation and institutional requirements. Written informed consent for participation was not provided by the participants’ legal guardians/next of kin because the data in the study was extracted from the SEER database, and the authors have obtained the approval to use the database.

## Author Contributions

YH, XM, and ZyL designed the study. YH, BjL, and XM extracted and analyzed the data. YH and BjL wrote and edited the manuscript. All authors contributed to the article and approved the submitted version.

## Conflict of Interest

The authors declare that the research was conducted in the absence of any commercial or financial relationships that could be construed as a potential conflict of interest.
