# Supplementary material for: Histological Characteristics and Early-Stage Diagnosis Are Associated With Better Survival in Young Patients With Epithelial Ovarian Cancer: A Retrospective Analysis Based on Surveillance Epidemiology and End Results Database
Source: Front Oncol. 2020 Dec 23;10:595789. doi: 10.3389/fonc.2020.595789 (PMC7787102; doi:10.3389/fonc.2020.595789)
Supplement: Supplementary file 1 [file DataSheet_1.pdf]

Supplementary Material

1 Supplementary Tables

Table S1 The association between histological type and AJCC stage in young and old patients with epithelial ovarian cancer in 2004-2015, SEER 18 registries.

		Patients ＜35 years (n=1,015)				Patients ⩾60 and ⩽79 years (n=15,833)			
		Ⅰ	Ⅱ	Ⅲ	Ⅳ	Ⅰ	Ⅱ	Ⅲ	Ⅳ
Histology ICD-O3	High-grade serous	50(18.5%)	18(6.7%)	152(56.3%)	50(18.5%)	978(9.0%)	992(9.2%)	6,101(56.4%)	2738(25.3%)
	Low-grade serous	19(24.4%)	7(9.0%)	47(60.3%)	5(6.4%)	101(33.0%)	34(11.1%)	145(47.4%)	26(8.5%)
	Endometrioid	149(80.1%)	23(12.4%)	13(7.0%)	1(0.5%)	768(70.5%)	162(14.9%)	132(12.1%)	27(2.5%)
	Mucinous	279(85.3%)	10(3.1%)	24(7.3%)	14(4.3%)	428(65.4%)	49(7.5%)	113(17.3%)	64(9.8%)
	Clear cell	25(67.6%)	3(8.1%)	7(18.9%)	2(5.4%)	404(55.5%)	83(11.4%)	178(24.5%)	63(8.7%)
	Carcinosarcoma	3(60.0%)	0	1(20.0%)	1(20.0%)	24(8.0%)	45(15.0%)	164(54.7%)	67(22.3%)
	Malignant Brenner	28(53.8%)	4(7.7%)	11(21.2%)	9(17.3%)	133(12.6%)	108(10.3%)	424(40.3%)	388(36.8%)
	Mixed	33(55.0%)	7(11.7%)	16(26.7%)	4(6.7%)	246(27.5%)	148(16.6%)	363(40.6%)	137(15.3%)

Table S2 Adjusted standardized value of post hoc test for clinicopathologic characteristics and surgery details in young and old patients with epithelial ovarian cancer in 2004-2015, SEER 18 registries.

		Patients ≥60 and ≤79 years (n=17,882)	Patients <35 years (n=1,071)
Race/ethnicity	White	7.8	-7.8
	Black	0.4	-0.4
	Asian or Pacific Islander	-10.6	10.6
	Others or unknown	-1.7	1.7
Histology ICD-O3	High-grade serous	27.1	-27.1
	Low-grade serous	-11.9	11.9
	Endometrioid	-13.4	13.4
	Mucinous	-37	37
	Clear cell	1.4	-1.4
	Carcinosarcoma	3.2	-3.2
	Malignant Brenner	1.9	-1.9
	Mixed	-0.4	0.4
Laterality	Right-origin primary	-6.5	6.5
	Left-origin primary	-8.1	8.1
	Paired site,but no information of laterality	4.1	-4.1
	Bilateral, single primary	11.2	-11.2
	Only one side-side unspecified	2.6	-2.6
AJCC Stage	I	-28.6	28.6

	II	3.2	-3.2
	III	13.3	-13.3
	IV	10.3	-10.3
CA125	Positive/elevated	10.8	-10.8
	Borderline	-1.7	1.7
	Nagetive/normal	-7.4	7.4
	Results unknown	0.9	-0.9
	Test not done	-8.6	8.6
	Unknown if the test did or not	-2.2	2.2
Reason of surgery not done	Surgery performed	-4.2	4.2
	Surgery not recommended/contraindications	4.2	-4.2
	Patients died before planned surgery	0.8	-0.8
	Patients or their relatives refused	-1.3	1.3
	Unknown	1.3	-1.3

Statistical significance is considered when the adjusted standardized value > 3

2 Supplementary Figures

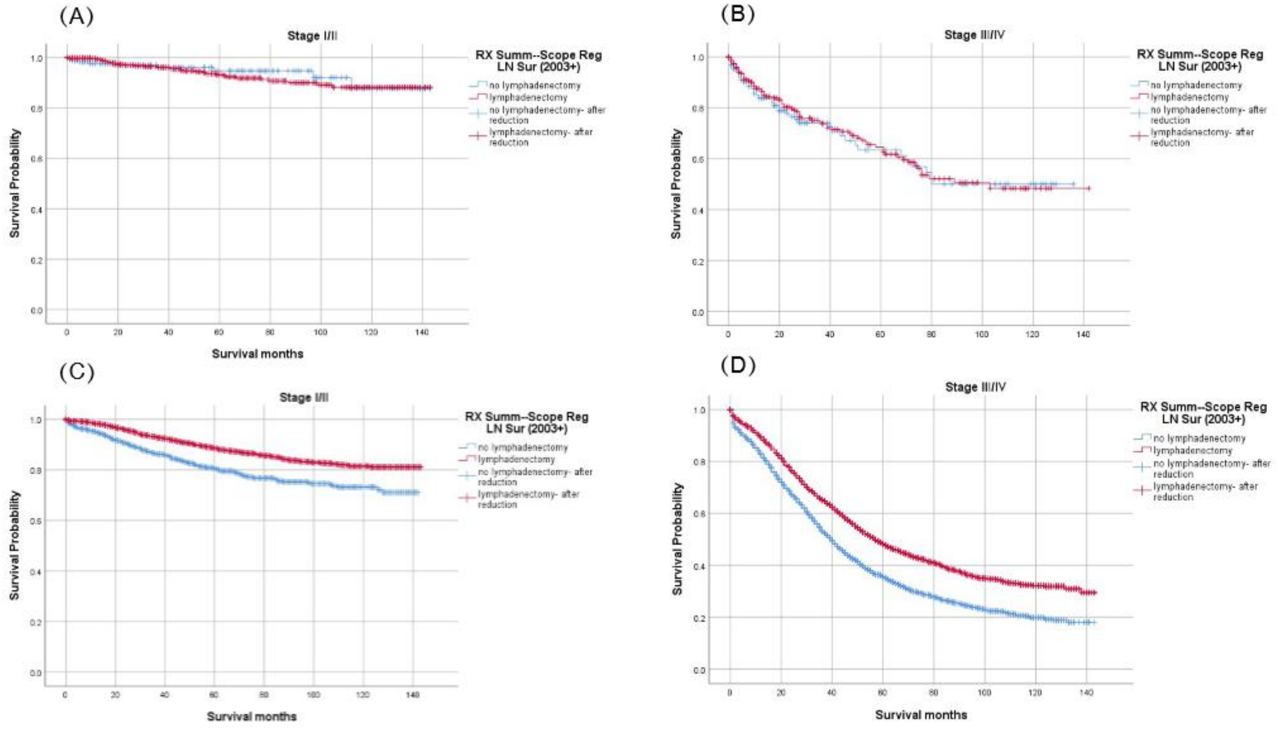

Supplementary Figure 1. Cause-specific survival on lymphadenectomy by stage in women aged< 35 and aged 60 to 79 with epithelial ovarian cancer. (a) Lymphadenectomy in women aged < 35 diagnosed at stage I/II; (b) Lymphadenectomy in women aged < 35 diagnosed at stage III/IV; (c) Lymphadenectomy in women aged 60-79 diagnosed at stage I/II; (d) Lymphadenectomy in women aged 60 to 79 diagnosed at stage III/IV.
